# Time‐lapse cameras reveal latitude and season influence breeding phenology durations in penguins

**DOI:** 10.1002/ece3.4160

**Published:** 2018-07-23

**Authors:** Caitlin Black, Ben Collen, Daniel Lunn, Dick Filby, Stephanie Winnard, Tom Hart

**Affiliations:** ^1^ Department of Zoology University of Oxford Oxford UK; ^2^ Department of Zoology University of Cambridge Cambridge UK; ^3^ Centre for Biodiversity & Environment Research University College London London UK; ^4^ Department of Statistics University of Oxford Oxford UK; ^5^ Rare Bird Alert Norwich UK; ^6^ British Antarctic Survey Cambridge UK; ^7^ Royal Society for the Protection of Birds Sandy UK

**Keywords:** annual cycle, Antarctica, chinstrap, gentoo, incubation, polar, *Pygoscelis*, seabird

## Abstract

Variation in the phenology of avian taxa has long been studied to understand how a species reacts to environmental changes over both space and time. Penguins (*Sphenicidae*) serve as an important example of how biotic and abiotic factors influence certain stages of seabird phenology because of their large ranges and the extreme, dynamic conditions present in their Southern Ocean habitats. Here, we examined the phenology of gentoo (*Pygoscelis papua*) and chinstrap penguins (*Pygoscelis antarctica*) at 17 sites across the Scotia arc, including the first documented monitoring of phenology on the South Sandwich Islands, to determine which breeding phases are intrinsic, or rather vary across a species range and between years. We used a novel method to measure seabird breeding phenology and egg and chick survival: time‐lapse cameras. Contrary to the long‐standing theory that these phases are consistent between colonies, we found that latitude and season had a predominant influence on the length of the nest establishment, incubation, and guard durations. We observe a trend toward longer incubation times occurring farther south, where ambient temperatures are colder, which may indicate that exposure to cold slows embryo growth. Across species, in colonies located farther south, parents abandoned nests later when eggs were lost or chicks died and the latest record of eggs or chicks in the nest occurred earlier during the breeding period. The variation in both space and time observed in penguin phenology provides evidence that the duration of phases within the annual cycle of birds is not fundamental, or genetic, as previously understood. Additionally, the recorded phenology dates should inform field researchers on the best timing to count colonies at the peak of breeding, which is poorly understood.

## 1 INTRODUCTION

Interspecific variation in the phenology of avian taxa has long been studied to understand a species’ basic biology and how a species reacts to environmental changes over both space and time (Schwartz, 2013). Variation mostly stems from an individual's requirement to match the peak in local resource quantity and quality to the peak of their own needs and the demands of their young (Visser & Both, 2005). In turn, phenology impacts reproductive success by dictating clutch size, egg mass, chick growth, and the likelihood of predation, making it relevant to population dynamics and individual fitness (Schwartz, 2013). Seabird phenology is particularly well studied because of their colonial nature and because the stages of seabird annual cycles are often highly constrained and synchronous (Gaston, 2004). Penguins (*Sphenicidae*) serve as an important example of how biotic and abiotic factors influence certain stages of seabird phenology because of their large ranges and the extreme conditions present in their Southern Ocean habitats (Black, 2015).

In Antarctic and sub‐Antarctic penguins, the variables dictating changes to the annual cycle differ significantly depending on the species and breeding site location (Black, 2015). In particular, variation in sea ice extent, especially prolonged pack ice, can delay a penguin's return to the breeding site and subsequently alter the timing of later breeding dates (Trivelpiece, Trivelpiece, & Volkman, 1987). Similarly, annual changes in food availability contribute to delayed breeding as adults must build up body condition and fat reserves prior to egg laying (Viñuela et al., 1996). Additional environmental factors, including wind conditions (Ainley & Leresche, 1973), sea surface temperature (Bost & Jouventin, 1990), and ambient air temperatures (Lynch, Fagan, Naveen, Trivelpiece, & Trivelpiece, 2012), can also impact the timing of breeding and the subsequent fitness of individuals. Likewise, abiotic factors, including the experience (Trivelpiece, Trivelpiece, & Volkman, 1984), health (Moreno, De Leon, Fargallo, & Moreno, 1998), and age (LeResche & Sladen, 1970) of adults, also dictate the breeding schedule. In addition, colony size (Barbosa, Moreno, Potti, & Merino, 1997), the sex of chicks (Fargallo et al., 2006), and nest location within a breeding site (Fargallo et al., 2006; MartÍn & Soler, 2006), significantly influence hatching dates, one of the most well‐studied periods in penguin phenology. Lastly, the timing of adult molt, which subsequently affects when chicks are left unguarded by their parents (Penteriani, Vinuela, Belliure, Bustamante, & Ferrer, 2003), is often triggered by changes in photoperiod (Ainley, 2002) and hormone levels (Groscolas, Jallageas, Goldsmith, & Assenmacher, 1986). Together, these variables, both environmental and individualistic, greatly affect certain stages of penguin phenology, yet many phases within the breeding cycle are considered fundamental (Borboroglu & Boersma, 2013), without variability throughout a species range and between years.

Certain periods within the breeding season are thought to be intrinsic across a species range, ingrained within the biology of the species rather than influenced by external variables. In gentoo penguins, *Pygoscelis papua*, these intrinsic parameters include a 2‐week nest attendance period prior to egg laying, a laying interval of approximately 3 days between eggs, an incubation period of 33–37 days, and general chick growth patterns (Bost & Jouventin, 1991). Similarly, in chinstrap penguins, *Pygoscelis antarctica*, laying intervals have been observed as 4 days (Lishman, 1985), with incubation lasting 33–36 days, and the guard period taking approximately 4 weeks (Borboroglu & Boersma, 2013). To date, no studies have, to our knowledge, found significant variability in any of these periods; however, past studies have not addressed why certain phases are plastic in their timing while others are ingrained in the biology of a species.

Here, we examined the phenology of gentoo and chinstrap penguins to fill in gaps in our understanding of each of the dates within their annual cycles and to determine which phases are indeed intrinsic or rather vary across a species range and between years. In particular, we aimed:

1. To establish the breeding phenology of two species of *Pygoscelis* penguins in the Southern Ocean, including all described phases (e.g., incubation, guard, and postguard).
2. To determine how the timing and duration of individual phases change along a latitudinal gradient and varies between years.
3. To understand how chick survival and nest abandonment rates are linked with phenology dates and durations to better comprehend the role phenology plays on individual fitness.

## 2 METHODS

### 2.1 Study sites

**Figure 1:**
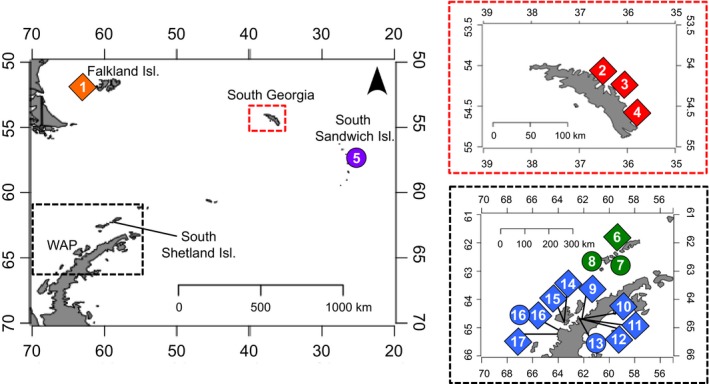
Map of 17 breeding site locations. Diamonds represent locations of gentoo penguin (*Pygoscelis papua*) colonies and circles represent locations of chinstrap penguin (*Pygoscelis antarctica*) colonies. The colonies are grouped by location from the Falklands Islands (orange), South Georgia (red), South Sandwich Islands (purple), South Shetland Islands (green), and the Western Antarctic Peninsula (blue). The numbers indicate each of the colonies specific location from (1) Beaver Island, (2) Maiviken, (3) Ocean Harbour, (4) Cooper Bay, (5) Saunders Island, (6) Aitcho Islands, (7) Half Moon Island, (8) Bailey Head, (9) Cuverville Island, (10) Georges Point, (11) Danco Island, (12) Spigot Peak, (13) Neko Harbour, (14) Damoy Point, (15) Port Lockroy, (16) Booth Island, and (17) Petermann Island

We monitored gentoo penguins at 13 sites and chinstrap penguins at four sites, ranging from the Falklands Islands, South Georgia, South Sandwich Islands, South Shetland Islands, and the Western Antarctic Peninsula (Figure 1). Study sites were chosen based on a nested design; we observed multiple breeding sites within a region and installed more than one camera at several breeding sites (Maiviken, Neko Harbour, Booth Island). Phenological dates had only been studied at three of the 17 study sites in the past (Bailey Head, Deception Island, Conroy, White, Furse, & Bruce, 1975; Viñuela et al., 1996; Barbosa et al., 1997; Moreno et al., 1998; Port Lockroy, Cobley & Shears, 1999; and Petermann Island, Gain, 1914). Colony counts for each study site during the study years can be found in Humphries et al. (2017).

### 2.2 Camera system

Twenty time‐lapse cameras were deployed at 17 sites beginning on 12 October 2012 to 24 February 2016 (Table 1). Not all cameras photographed colonies during the same dates due to the logistical difficulties of setting up cameras on the same date across the two species’ ranges (Table 1). Each camera was installed roughly three meters from nesting subcolonies at each of our study sites. The cameras were installed using techniques similar to those described by Newbery and Southwell (2009) and Southwell and Emmerson (2015), with minor adjustments to the camera system, as reported in Black, Collen, Johnston, and Hart (2016). At each site, a Reconyx HC500 Hyperfire trail camera (Reconyx, Inc., Holmen, WI, USA) was mounted to a scaffold pole and anchored using a rock basket (Antarctic Peninsula, South Shetland Islands, and South Sandwich Islands) or by being hammered into tussock grass or soil (Falklands Islands and South Georgia). The location of each camera was dictated by the distance from nearby nesting sites but also determined opportunistically using locations with higher elevation, appropriate substrate (rock or tussock grass) for anchoring the camera system, and low snow accumulation (Southwell & Emmerson, 2015). The cameras were programmed in time‐lapse mode to take six photographs daily during year‐long peak daylight hours at 1000, 1100, 1200, 1300, 1400, and 1500 local time, and each camera captured images of roughly 15 nests. Once installed, the cameras were maintained yearly to retrieve data from the previous year, check operations, and change the batteries. In a few cases, the camera angle did change year to year due to alterations in the location of nesting subcolonies (Booth Island, Georges Point, Maiviken, and Petermann Island), which is common in gentoo penguins. In addition, to determine the accuracy of our methods using six images daily, we attached the same camera system to the same pole at five sites (Cooper Bay, Georges Point, Neko Harbor, Port Lockroy, and Booth Island) but changed the camera frequency setting to photograph the same nests every minute instead of every hour.

**Table 1:** A summary of the breeding site locations and data available for gentoo (*Pygoscelis papua*) and chinstrap penguins (*Pygoscelis antarctica*), including latitudes, longitudes, and dates of operation

Species	Region	Site	Latitude, Longitude	Observation dates for each camera
Gentoo	Falkland Isl.	Beaver Isl.	51°50′S, 61°16′W	7 December 2014–6 September 2015
	South Georgia Isl.	Cooper Bay	54°48′S, 35°47′W	26 December 2014–28 October 2015
		Maiviken	54°14′S, 36°29′W	12 October 2012–2 February 2015
				15 October 2012–2 January 2015
		Ocean Harbour	54°20′S, 36°16′W	31 October 2014–12 November 2015
	South Shetland Isl.	Aitcho Isl.	62°23′S, 59°46′W	9 January 2014–5 December 2015
	Western Antarctic Peninsula	Booth Isl.	65°03′S, 64°01′W	3 December 2012–29 December 2015
		Cuverville Isl.	64°41′S, 62°38′W	10 January 2014–6 January 2016
		Damoy Point	64°81′S, 63°53′W	26 December 2012–20 November 2014
		Danco Isl.	64°43′S, 62°35′W	5 December 2012–6 December 2015
		Georges Point	64°40′S, 62°40′W	22 December 2012–6 January 2016
		Neko Harbour	64°50′S, 62°31′W	3 March 2012–6 January 2016
				14 December 2012–6 January 2016
		Petermann Isl.	65°10′S, 64°08′W	12 December 2012–8 January 2016
		Port Lockroy	64°49′S, 63°29′W	13 December 2012–7 January 2016
Chinstrap	South Sandwich Isl.	Saunders Isl.	57°47′S, 26°27′W	12 November 2013–24 February 2016
	South Shetland Isl.	Bailey Head	62°58′S, 60°30′W	20 December 2012–20 November 2014
		Half Moon Isl.	62°59′S, 59°92′W	12 December 2012–24 December 2015
				21 December 2012–11 December 2015
	Western Antarctic Peninsula	Booth Isl.	65°03′S, 64°01′W	3 December 2012–29 December 2015
		Spigot Peak	64°37′S, 62°33′W	25 November 2012–9 February 2016

### 2.3 Phenological dates

Each nest at each location was monitored from 12 October 2012 to 24 February 2016, where data were available (see Table 1 for range of dates, which vary by location). For either each nest (nesting, laying of 1st egg aka. clutch initiation, laying of 2nd egg, hatching of 1st and 2nd eggs, guard and postguard phases) or for the entire breeding site (arrival and departure dates, adult and chick molt dates), the following dates were recorded from images.

1. Arrival (breeding site wide): In chinstraps only, we noted the first day when individuals appear at the breeding site because individuals are not present at the breeding site continuously over winter. Because gentoo individuals are present year‐round at the breeding site (Bost & Jouventin, 1991; Black et al., 2017), we did not define arrival date.
2. Nesting (individual nests): At each nest, we noted the date when partners first began sitting on a nest continuously for a 24‐hr period. The nesting duration began at the first nesting date and ended when the 1st egg was laid. During this period, birds are often seen building nests and copulating.
3. Laying of 1st egg (individual nests): At each nest, we noted the date when an egg was first observed.
4. Laying of 2nd egg (individual nests): At each nest, we noted the date when a 2nd egg was first observed.
5. Laying interval (individual nests). At each nest, we noted the number of days between the date when the 1st egg was laid and the 2nd egg was laid.
6. Incubation duration (individual nests): At each nest, the incubation duration began when the 1st egg was laid and ended when the 1st chick hatched.
7. Hatching of 1st chick (individual nests): At each nest, we noted the date when a 1st chick was first observed.
8. Hatching of 2nd chick (individual nests): At each nest, we noted the date when a 2nd chick was first observed.
9. Hatching interval (individual nests). At each nest, we noted the number of days between the date when the 1st chick hatched and the 2nd chick hatched.
10. Guard duration (individual nests): We considered the guard period to begin when the 1st chick was first observed (see #5) and end when the 1st chick left was left unguarded (see Black et al., 2016 for definition of parental guard) for 24 hr (aka. guard end date).
11. Adult molt (breeding site wide): We noted the first and last dates when adults were observed molting with clearly displaced feathers.
12. Chick molt (breeding site wide): We noted the first date when chicks were viewed molting. The end of chick molt was not noted because chicks fledged during molt.
13. Adult departure (breeding site wide): In chinstraps only, we noted the last date when adults were observed at the nesting site for a period of at least 15 days after molting. Because individual gentoos are present year‐round at the breeding site (Bost & Jouventin, 1991; Black et al., in review), we did not defined the departure date.

We must note that the dates observed from time‐lapse cameras have been found to be 0–2 days (mean = 0.9 days) later for first arrival dates and 2–6 days later for first egg laying (Southwell & Emmerson, 2015).

### 2.4 Nest survival rates

Chick survival was monitored at each nest at each site during the study period. We noted whether individuals at each nest raised 0, 1, or 2 chicks to the end of the guard phase (see definition above, CCAMLR Standard Method A6B) of each year and, whether fewer than two chicks survived to the beginning of the postguard phase, we noted the dates when the last chick or egg was seen at the nest (last seen date; Southwell & Emmerson, 2015). In addition, we noted whether individuals in each nest laid 0, 1, or 2 eggs (e.g., total eggs) and whether 0, 1, or 2 chicks hatched (e.g., total chicks). We also noted whether nests were abandoned and the date of abandonment; abandonment was defined as the last day a parent was seen on the nest prior to the end of the postguard phase.

### 2.5 Statistical analysis

All analyses were conducted in R (v 3.1.3) language for statistical computing (R Core Development Team 2013). Survival analysis was conducted using the *Surv* and *survreg* functions in the *survival* package (Therneau, 2015). We chose the following parametric survival regression models instead of Cox proportional hazards regression models due to left‐censored data (when the start rather than end date is unknown) in four of our models (those with nest duration, laying interval, incubation duration, hatching interval, and guard duration as response variables), which are not possible using Cox proportional hazards regression models. Initially, breeding site was added as a random effect to each of our survival models but latter removed because the random effect did not account for a large percent of the variation in the model and latitude was instead used as a substitute for breeding site location. The remaining two models (last seen date and nest abandonment) were right‐censored in our survival analysis. For each of the models, we fitted Weibull, exponential, Gaussian, logistic, lognormal, and log logistic distributions and chose the distribution with the highest log‐likelihood (Table 2). Due to a large number of missing values (as a result of the timing of camera placement, snowdrift obstructing the view, and the difficulty of observing both egg‐laying events) and to avoid overfitting the model, nesting start date, nesting duration, and laying interval were not used as explanatory variables in any of the models (Table 2).

**Table 2:** Summary of survival models used, including the distribution, log‐likelihood, total observations in data set (*n*), and the variables removed

Response variable	Candidate explanatory variables	Model simplification	Final model	Distribution	Log‐likelihood	*n*
Nest duration	Species, Latitude, and Season	N/A	Nest duration—Species, Latitude, and Season	Weibull	−361	162
Laying interval	Species, Latitude, and Season	Latitude (*p* = .62) Species (*p* = .89)	Laying interval—Season	Weibull	−55.1	33
Incubation duration	Species, Latitude, and Season	N/A	Incubation duration—Species, Latitude, and Season	Gaussian	−433.1	334
Hatching interval	Species, Latitude, Incubation duration, and Season	Latitude (*p* = .93) Incubation duration (*p* = 1)	Hatching interval—Species and Season	Weibull	−369.5	222
Guard duration	Species, Latitude, Season, Incubation duration, Hatching interval, Adult molt start date, and Chick molt start date	Incubation duration (*p* = 1)	Guard duration—Species, Latitude, Season, Hatching interval, Adult molt start date, and Chick molt start date	Log normal	−761.5	281
Last seen date	Species, Latitude, and Season	Season (*p* = .59)	Last seen date—Species and Latitude	Weibull	−417.6	186
Nest abandonment date	Species, Latitude, Egg total, Chick total, and Season	Season (*p* = .61)	Nest abandonment date—Species, Latitude, Egg total, and Chick total	Weibull	−374.3	158

Next, to examine influences on nest was abandoned, we fitted a binomial generalized linear mixed model (GLMM) using the *glmmPQL* function in the *MASS* package (Venables & Ripley, 2002). The breeding site locations (e.g., Aitcho Island) were considered a random effect in the model. The residuals of the model were then graphed using box‐and‐whisker plots to examine the differences in nest abandonment across breeding site locations (Figure S1). Due to a large number of missing values and to avoid overfitting the model, nesting start date, nesting duration, first laying date, incubation duration, and laying interval were not used as explanatory variables in the following model (*n* = 426).

### 2.6 Nest abandonment (binary)—species, season, chick total

**Figure 2:**
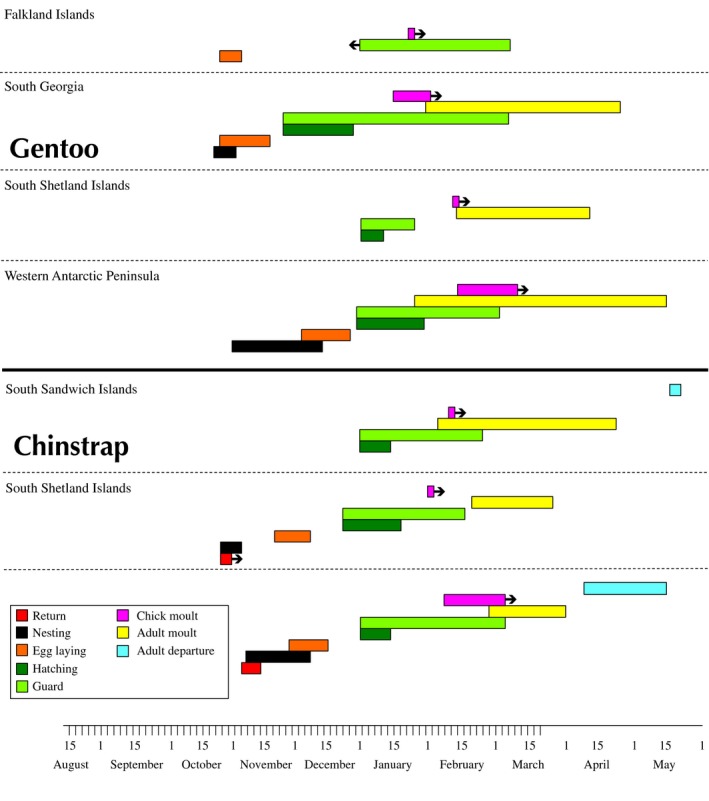
Gantt chart summarizing dates and duration of each phenological phase during the breeding period by location (Falkland Islands, South Georgia, South Sandwich Islands, South Shetland Islands, and the Western Antarctic Peninsula (WAP)) and species (gentoo, *Pygoscelis papua,* and chinstrap, *Pygoscelis antarctica* penguins)

To examine the variation and synchronicity of three particular dates (first laying, first hatching, and guard end dates) across all 17 breeding sites, we graphed the 95% confidence intervals of each date at each site using the *plotCI* function in the *gplots* package (Warnes et al., 2013; Figure 2). In order to create this graph, we first transformed all three dates by raising them to the power of 8 (after examining the skew of data to the power of 2 and 4) because, when plotting the density of each of the dates, the data were left‐skewed. We then adjusted the dates by season to account for seasonal variation within the colonies.

Lastly, to determine the accuracy of our methods, we compared the hatching dates obtained from cameras taking six photographs daily to those taking a photograph every minute at a subset of sites (Cooper Bay, Georges Point, Neko Harbor, and Booth Island). From the images taken every minute, we then obtained the 1st hatching dates, as described above, using the same methods. We compared the hatching dates obtained from the cameras taking six photographs daily to those taking photographs every minute by undergoing a paired samples *t* test for using the *t.test* function in the *stats* package (A 2013).

## 3 RESULTS

### 3.1 Phase durations

When examining the duration of phases within the breeding period across sites and years in both gentoo and chinstrap penguins, we found that latitude and season influenced the length, in days, of the nesting duration (first nesting activity to first egg laid), incubation duration (first egg laid to first chick hatched), and guard duration (first chick hatched to the last date both chicks seen guarded, when applicable; Table 3). In addition, in all models (with the exception of laying interval, where we did not find any significant variables in our model), species affected the duration (Table 3). Specifically, chinstraps exhibited longer nesting and hard durations and hatching intervals, and guard durations than gentoo penguins, while the opposite held true with incubation duration (Table 3). Nest establishment duration increased as latitude decreased; in other words, colonies located farther north underwent more time between first nesting and laying eggs (Table 3). The opposite held true with incubation and guard durations; colonies located farther south incubated eggs longer and guarded their chicks for more days (Table 3). Lastly, the breeding site wide start date of the adult molt significantly influenced the guard duration; when the adult molt started later, the guard duration was longer (Table 3).

**Table 3:** Summary of model outputs as a result of survival analyses and a binomial generalized mixed model. Output from survival model of laying interval not included in table because results revealed no significant explanatory variables

Analysis	Response variable	Explanatory variable		Value	Standard error	*p*
Survival	Nesting duration	Species	Chinstrap^a^	2.065	1.181	.08
			Gentoo	−0.776	0.112	<.001***
		Latitude		−0.043	0.018	.02*
		Season	2013–2014	4.070	0.108	<.001***
			2014–2015	4.228	0.000	<.001***
Survival	Incubation duration	Species	Chinstrap^a^	−114.8	28.06	<.001***
			Gentoo	20.37	3.658	<.001***
		Latitude		1.72	0.435	<.001***
		Season	2013–2014	−1.54	3.233	.64
			2014–2015	15.71	3.435	<.001***
Survival	Hatching interval	Species	Chinstrap^a^	0.918	0.163	<.001***
			Gentoo	−0.181	0.166	.28
		Season	2013–2014	0.590	0.172	<.001***
			2014–2015	0.233	0.183	.20
Survival	Guard duration	Species	Chinstrap^a^	1.099	0.581	.06
			Gentoo	−0.176	0.041	<.001***
		Latitude		0.012	0.004	<.01**
		Season	2013–2014	0.177	0.036	<.001***
			2014–2015	0.189	0.045	<.001***
		Adult molt start date		0.004	0.001	<.01**
Survival	Last seen date	Species	Chinstrap^a^	5.633	0.055	<.001***
			Gentoo	0.003	0.009	.73
		Latitude		0.005	0.001	<.001***
Survival	Nest abandonment date	Species	Chinstrap^a^	5.546	0.068	<.001***
			Gentoo	0.001	0.010	.91
		Latitude		0.006	0.001	<.001***
		Egg total		0.052	0.011	<.001***
		Chick total		−0.014	0.007	.04*
Binomial generalized mixed model	Nest abandonment (binary)	Species	Chinstrap^a^	1.505	1.116	.18
			Gentoo	1.912	0.837	.02*
		Season	2013–2014	−1.898	0.421	<.001***
			2014–2015	0.036	0.567	.95
		Chick total		1.336	0.191	<.001***

^a^Reference level for each response variable.

*p < .04, **p < .01, ***p < .001.

Annual effects were significant (*p* < .001); longer nesting, incubation, and guard durations occurred in the 2014–2015 season compared to the summer of 2013–2014 (Table 3). However, hatching intervals were longer in the 2013–2014 season than the 2014–2015 season (Table 3).

### 3.2 Survival of eggs and chicks

Survival analysis on the dates when either an egg or chick was last seen in the nest before loss or death and the dates when parents abandoned their nests after eggs or chicks disappeared revealed distinct differences between species (Table 3). Chinstrap penguins abandoned the nest significantly later, and their eggs or chicks were last seen later in the breeding season than gentoo penguins (Table 3). In addition, across species, colonies located farther south (higher latitudes) abandoned nests later and eggs or chicks were last seen in the nest at advanced dates in the breeding period (Table 3). Furthermore, as the total number of eggs laid in each nest increased, the date of abandonment advanced (Table 3).

### 3.3 Nest abandonment

The binomial generalized mixed model on survival revealed that (1) gentoos were more likely to abandon a nest than chinstraps, (2) there were significantly less nests abandoned in the 2013–2014 season than in the 2014–2015 season, and (3) when more chicks were in the nests, parents were more likely to abandon the nest (Table 3). When examining the residuals of the random effect, breeding site, used in our model, we found that three breeding site locations were unique in their abandonment rates, all of which are inhabited by chinstrap penguins: (1) Saunders Island, South Sandwich Islands, (2) Half Moon Island, South Sandwich Islands, and (3) Booth Island, WAP (Figure S1). Saunders and Booth Islands showed large variation in their abandonment rates with a slightly higher than average mean, while both cameras at Half Moon Island observed much lower than average abandonment rates (Figure S1).

### 3.4 Phenology dates

When observing the 95% confidence intervals at each site for the dates of first egg laying, first hatching, and the end of the guard period, we found differences between two distinct regions for each of these dates (Figure 2). For the first laying and first hatching dates, South Georgia colonies laid and hatched eggs significantly earlier than those in the South Sandwich Islands, South Shetlands Islands, and WAP (Figure 2). For the end date of the guard period, the Falkland Island, South Georgia, and the South Shetland Island birds appeared to end the guard period earlier, albeit not significantly, than birds in the South Sandwich Islands and WAP (Figure 2).

### 3.5 Camera accuracy

Lastly, our paired sample *t* test of the first hatching dates as observed by cameras taking six photographs daily vs. cameras taking a photograph every minute daily showed no significant differences between the two groups (*t* = −0.91, *df* = 16, *p* = .38). In other words, increasing the frequency of photographs taken from six daily to every minute did not significantly influence the dates observed.

Anecdotally, one replacement clutch occurred at Neko Harbour during the 2014–2015 season, although neither egg hatched. In addition, in one nest at Half Moon Island during the 2014–2015 season, three eggs were laid and all eggs hatched; however, all three chicks eventually died.

## 4 DISCUSSION

We provide evidence that the duration of key phases, thought to be intrinsic within these two species (Borboroglu & Boersma, 2013; Bost & Jouventin, 1991; Lishman, 1985), is instead highly plastic between years and vary along a latitudinal gradient. Most surprisingly, for the first time in any avian taxa documented in situ, we found that individuals nesting farther south incubated their eggs longer. In addition, individuals nesting in colonies farther south exhibited shorter nesting periods prior to egg laying but guarded their chicks longer. We also found latitudinal differences in the timing of nest abandonment, demonstrating how these phenology shifts influence individual fitness at the breeding sites studied. By examining numerous sites across the species’ ranges, documenting a large sample size of individual nests, and analyzing variation across multiple seasons, we provide evidence that avian phenology has more trade‐offs between breeding stages, years, and species than previously recognized.

It is well established that clutch size and nest attentiveness can vary with latitude (Deeming, 2002), yet, to our knowledge, we are the first to discover interspecific variability in incubation duration in avian taxa using field‐based methods. A past study, taking eggs from subspecies (temperate, *Troglodytes aedon aedon*, and tropical, *Troglodytes aedon musculus*, house wrens) at two locations and incubating them in a laboratory, found that chicks from eggs located farther south hatched on average 1.2 days later than the other, more equatorial subspecies (Robinson, Styrsky, Payne, Harper, & Thompson, 2008). Both meta‐analyses examining intraspecific relationships in incubation duration (Martin, Auer, Bassar, Niklison, & Lloyd, 2007; Martin & Schwabl, 2008) and laboratory experiments exposing eggs to a variety of conditions (Olson, Vleck, & Vleck, 2006) have found that when eggs are exposed to colder temperatures, chicks consequently take longer to hatch and adults produce larger eggs (Martin, 2008). The gentoo colonies studied here nest on a variety of substrates (e.g., bare soil in the Falkland Islands, Tussock grass in South Georgia, and rocks in all other locations), which may explain differences in exposure to ambient temperature and therefore the spatial variation observed; however, the chinstrap colonies we studied nest exclusively on rocks at all study sites yet also varied significantly in the length of incubation across a latitudinal gradient. Laboratory‐based experiments have also provided evidence that increased daylight hours can speed up embryo growth within an egg, shortening the length of incubation (Cooper, Voss, Ardia, Austin, & Robinson, 2011; Isakson, Huffman, & Siegel, 1970; Shutze, Lauber, Kato, & Wilson, 1962). The trend we observe here, in penguins, toward longer incubation times farther south, where air temperatures are colder and summer daylight hours during the breeding period are longer, likely indicates that exposure to cold temperatures outweighs any photoacceleration in these two species. Additional studies on intraspecific variation in incubation duration have also revealed that high predation, small body size, and high‐quality food resources may speed up incubation times, which may also explain some of the variation seen here in the pygoscelids (Boersma, 1982; Bosque & Bosque, 1995; Krebs & Avery, 1984; Lack, 1968; Nice, 1954; Perrins, 1976; Ricklefs, 1968; Worth, 1940). Future studies should focus on responses to these variables in situ within one species.

Colonies located farther south also exhibited significantly shorter nesting periods but guarded their chicks longer. The shift toward laying eggs shortly after beginning to build a nest at the breeding site may be a result of penguins shifting their phenology to provide maximum time for embryo growth within eggs in areas where cold exposure may stunt the growth rate. In both species, the date when adults molt influences how long the chicks are guarded, as parents, must trade off the need to build up energy reserves prior to molt (known as hyperphagia) while continuing to care for their young (Penteriani et al., 2003). The longer guard durations observed farther south may result from adults beginning their molt at later dates. Future studies should track individual molt times (rather than breeding site wide, as done here) to better understand latitudinal trends in molt and guard duration.

In addition, across species, in cases where loss or deaths occurred, colonies located farther south abandoned nests later and eggs or chicks were last seen in the nest at advanced dates in the breeding period (Table 3). This delay in egg or chick loss and nest abandonment may also be linked to the longer incubation durations observed farther south; as eggs take longer to fully develop before hatching, the chance of the egg succumbing to predation or spoiling in a flooded nest increases, leading to later abandonments and deaths. Our analyses also revealed that penguins were more likely to abandon a nest when more chicks were present; as two chicks are more costly to feed than one, particularly with the constraints of avoiding phenological mismatch, these demands increase the likelihood of parents not being able to adequately feed chicks, leading to abandonment. In addition, we found that gentoos are more likely to abandon a nest than chinstraps, although, when chinstraps do abandon, the eggs or chicks are more advanced in age. We also discovered that three breeding site locations exhibited unique trends in their abandonment rates, all of which are inhabited by chinstrap penguins: (1) Saunders Island, South Sandwich Islands, (2) Half Moon Island, South Shetland Islands, and (3) Booth Island, WAP (Figure S1). We provide further evidence of variability in chinstrap populations (Trivelpiece et al., 2011), where some colonies exhibit low nest abandonment (Half Moon Island, South Shetland Islands) while others show large deviations in how likely an individual's nest will fail (Saunders Island, South Sandwich Islands, and Booth Island, WAP).

**Figure 3:**
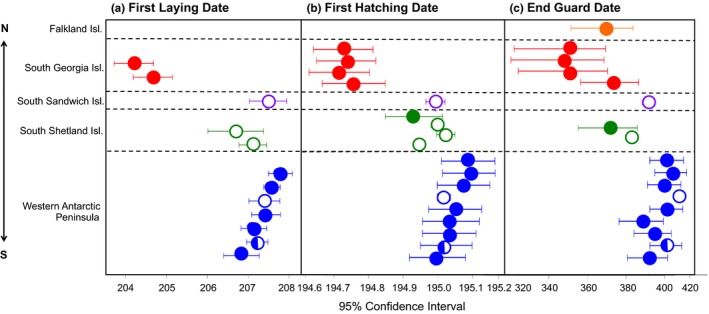
95% confidence interval plots of (a) first laying date, (b) first hatching date, and (c) guard end date at 17 breeding site locations for gentoo and chinstrap penguins. Colors indicate the following breeding site location: (1) Falkland Islands (orange), (2) South Georgia (red), (3) South Sandwich Islands (purple), (4) South Shetland Islands (green), and (5) Western Antarctic Peninsula (blue). Closed circles indicate sites where only gentoo penguins were observed, open circles indicate sites where only chinstrap penguins were observed, and the half‐closed circles indicate the site where both gentoos and chinstraps were observed. Latitude increases from top to bottom. Adjusted *R* ^2^ values and *df* included (a) *R* ^2^ = .99, *df* = 143, (b) *R* ^2^ = .99, *df* = 313, and (c) *R* ^2^ = .99, *df* = 331

The variation in both space and time observed here in penguin phenology provides evidence that the duration of phases within the annual cycle of birds is not intrinsic, or genetic, as previously understood. Here, we have successfully filled in gaps in the phenological dates of the two species to better understand their basic biology, particularly during the guard period and adult and chick molt (Figures 2 and 3). Furthermore, we have shown the applications of noninvasive time‐lapse cameras to the study of phenology and that even as few as six photographs daily can provide enough information to observe accurate dates at both breeding site and individual levels and examine spatial and temporal trends. The recorded phenology dates should also inform field researchers on the best timing to count colonies at the peak of breeding (Southwell, McKinlay, Emmerson, Trebilco, & Newbery, 2010) and thereby control for count differences due to surveying the birds during different phenological phases. Future research can improve upon these methods by observing more years and gaining information on the ages and experience of individuals. As climate change occurs within these species’ ranges, particularly along the rapidly warming Western Antarctic Peninsula (Mulvaney et al., 2012), understanding phenological shifts over time can help researchers to predict how species, and even specific colonies, respond to microclimatic changes.

## CONFLICT OF INTEREST

None declared.

## AUTHOR CONTRIBUTIONS

TH and BC funded the project. TH, BC, CB, SW, and DF collected the data. CB and DL underwent the statistical analysis. CB wrote the paper. All authors edited the paper.
