# Supplementary material for: Time‐lapse cameras reveal latitude and season influence breeding phenology durations in penguins
Source: Ecol Evol. 2018 Jul 23;8(16):8286–96. doi: 10.1002/ece3.4160 (PMC6144991; doi:10.1002/ece3.4160)
Supplement: Supplementary file 1 [file ECE3-8-8286-s001.docx]

**Supplementary Material**

### Appendix I. Anecdotes

Anecdotally, one replacement clutch occurred at Neko Harbour during the 2014- 2015 season, although neither egg hatched. In addition, in one nest at Half Moon Island during the 2014- 2015 season, three eggs were laid and all eggs hatched; however, all three chicks eventually died.

**Figure S.1**. Box-and-whisker plots of residuals resulting from binomial generalized mixed model of nest abandonment, showing difference in nest abandonment as a binary variable across 20 sub-colonies at 17 locations. Colours indicate the following colony location: 1) Falkland Islands (orange), 2) South Georgia (red), 3) South Sandwich Islands (purple), 4) South Shetland Islands (green), and 5) Western Antarctic Peninsula (blue). Diamonds indicate sites where only gentoo penguins were observed, circles indicate sites where only chinstrap penguins were observed, and the triangle indicates the site where both gentoos and chinstraps were observed. Latitude increases from left to right.
